# Inbreeding in the Zwartbles breed population and its influence on meat production in the Czech Republic

**DOI:** 10.5194/aab-66-245-2023

**Published:** 2023-09-13

**Authors:** Radek Filipčík, Daniel Falta, Dominika Sokolová, Martin Hošek, Vojtěch Pešan, Tomáš Kopec

**Affiliations:** Department of Animal Breeding, Faculty of AgriSciences, Mendel University in Brno, Zemědělská 1, 613 00 Brno, Czech Republic

## Abstract

The aim of this study was to estimate the inbreeding coefficient in the Zwartbles sheep population in the Czech Republic, as well as to investigate the effect of inbreeding on meat yield indicators. The analysis used data on the entire population since 1997, with the original database containing 13 150 animals. In this population, the average inbreeding coefficient is 3.64 %. There is a significant increase in inbreeding – from zero values to more than 4 % between 1997 and 2021. The effect of inbreeding on the weight of the animals at 100 d of age, the weight at bonitation prior to breeding, the thickness of the *musculus longissimus dorsi* (MLD), the thickness of the subcutaneous back fat, and the fleshiness of the animals were all evaluated. The value of the inbreeding coefficient had a statistically significant effect on weight at 100 d, MLD thickness, and back fat thickness. A negative relationship between inbreeding and all of these parameters was found when the inbreeding coefficient was increased by 1 %, resulting in a 60.2 g weight reduction at 100 d, 0.06 mm reduction in MLD thickness, and 0.013 mm reduction in back fat thickness.

## 1 Introduction

Meat sheep breeding is currently the most important sector of sheep breeding in the Czech Republic. Sheep of the Zwartbles breed are kept in small herds and are a less numerous but dynamically developing breed in the Czech Republic. Research on meat productivity in the Zwartbles breed in the Czech Republic has already been carried out by Komprda et al. (2012), who evaluated slaughter parameters and meat quality in the Zwartbles, Suffolk, and Oxford Down breeds.

A variety of factors influence meat yield, including animal individuality, housing, nutrition, and breed affiliation. These aspects have been discussed, for example, by McGovern et al. (2020) and Fetherstone et al. (2022). The influence of the management of reproduction, gender, and litter frequency on meat yield is also assessed (Ilic et al., 2013). The genetic foundation of meat yield and external influences on meat were evaluated, for example, in the D'man sheep breed (Boujenane et al., 2015).

The value of the inbreeding coefficient is another important factor influencing sheep meat productivity. It has negative effects on both milk yield (Cesarani et al., 2023) and meat yield (Kiya et al., 2019). Inbreeding has also been shown to have a negative effect on milk yield in goats, with a 1 % increase in the inbreeding coefficient resulting in a 2.31 kg decrease in milk yield per lactation (Paiva et al., 2020). In Denmark, the effect of inbreeding on meat yield was analyzed in three main meat sheep breeds (Norberg and Sorensen, 2007). The mentioned works describe the negative influence of inbreeding on the main parameters of sheep meat yield, particularly on the development of the *longissimus dorsi* muscle, the thickness of back fat, birth weight, and average daily gain in lambs. The inbreeding coefficient has a significant effect on sheep reproductive indicators. Inbreeding, for example, has been shown to reduce fertility, fecundity, and prolificacy in Leccese sheep in Italy (Selvaggi et al., 2010).

The structure of sheep populations and the estimation of the inbreeding coefficient in these populations have been discussed by many authors (Eteqadi et al., 2014; Kiya et al., 2019; Norberg and Sorensen, 2007). Its ever-increasing value, combined with a negative effect on a number of important production and reproductive traits, may cause issues in the future, similar to what we see in important dairy cattle breeds (da Silva et al., 2019). Between 2007 and 2011, only animals with an inbreeding coefficient of up to 5 % were found in the Dorper sheep population in Brazil. Following that, there is already an increase in animals with higher inbreeding. Over six generations, the average value of the inbreeding coefficient increased from nearly zero values to 1.65 % (Kiya et al., 2019). In Denmark, significant populations of meat sheep breeds have seen an increase in the inbreeding coefficient (Norberg and Sorensen, 2007). Population structure, size of inbreeding, and effective population size are also addressed in goats. The Saanen goat in Brazil has an average inbreeding coefficient of 1.48 % and is constantly increasing (Paiva et al., 2020).

With the recent development of modern breeding tools, it is now possible to assess the degree of inbreeding in sheep populations using data from animal genomic selection. In Switzerland, genomic data were used to assess inbreeding in sheep (Signer-Hasler et al., 2019). This can reveal differences in the inbreeding coefficient between full siblings and where they have an effect in the genome. This opens up new avenues for increasing genetic diversity within the breed. Information about genomic inbreeding can help us better understand population kinship relationships and make better decisions when organizing animal breeding. The use of genomic and animal pedigree information to improve inbreeding management in sheep and goat populations in Italy has demonstrated additional undeniable benefits of involving genomics in this issue (Cortellari et al., 2022).

The aim of the study was to determine the level of kinship in the Zwartbles breed population in the Czech Republic and to further assess the effect of this kinship on meat yield parameters. The inbreeding coefficient expresses kinship, and the null hypothesis states that it has no effect on meat production indicators.

## 2 Material and methods

The evaluation included all Zwartbles sheep in the Czech Republic that are involved in official animal recording of sheep. A total of 13 150 individuals of the Zwartbles breed born between 1997 and 2021 were evaluated in the pedigree. The inbreeding coefficient was calculated using all individuals. The current average size of the Zwartbles population involved in performance control is around 750 mature sheep. Because the breed is bred primarily for meat yield, the effects of the inbreeding coefficient on meat yield parameters were investigated. The influence on weight at 100 d of age (Weight100) as well as weight at bonitation before selection for breeding (Weight300), the thickness of *musculus longissimus* *dorsi* (MLD) in millimeters at 100 d, the thickness of back fat in millimeters (BackFat) at 100 d, and the fleshiness at 100 d were all evaluated (subjective point evaluation on a scale of 1–5). Body weight was measured using an individual digital weight scale for sheep (Superdamp™ technology, ISO 9001). These data are routinely collected during sheep performance recording in the Czech Republic. The apparatus method is used to determine BackFat thickness and MLD behind the last thoracic vertebra using the DP-20 VET (at the age of 100 d as part of official animal recording of sheep). Weight100 was recorded in 11 534 sheep, Weight300 in 2813 sheep, MLD thickness in 11 533 sheep, back fat thickness in 1731 sheep, and fleshiness in 1715 sheep. Weight100, MLD fatness, and BackFat were measured in animals aged 70–130 d and Weight300 at around 300 d (214–378 d).

The average farm size in the largest set for Weight100 was 16 animals, and animals from 78 farms were included in the calculation. Twins were the most common in the litter (63.01 %), with singletons accounting for 19.09 % of the total. Females constituted 53.29 % of the population.

Grazing production systems based on the efficient and sustainable use of permanent grasslands, with minimization of labor intensity and external inputs into the system, predominate in the Czech Republic. During the natural breeding season (August to November), breeding is mostly done in a harem style. Only rams who have undergone bonitation can be used for breeding. During the winter, the animals are kept in sheepfolds, and on some farms, lamb feeding is organized.

### Statistical analysis

The PROC INBREED procedure in SAS 9.1 software (SAS Institute, 2004) was used to calculate inbreeding coefficients. The effect of the level of the inbreeding coefficient on meat yield parameters was estimated using the GLM (general linear model) method using PROC GLM in SAS 9.1. software. In addition to the influence of inbreeding, the model included the following explanatory variables:

$$\begin{array}{rl} y_{ijklmn} & =\mu +b_{1}{\text{age}}_{i}+b_{2}{\text{age2}}_{i}+b_{3}F_{i}+{\text{sex}}_{j}+{\text{freq}}_{k}\\ & +{\text{breeder}}_{l}+{\text{month}}_{m}+{\text{year}}_{n}+e_{ijklmn}, \end{array}$$

where $y_{ijklmn}$ is the dependent variable (the selected indicator of meat yield); age and age2 denote the quadratic regression of age on meat yield indicators (age is the age of animals in days related to the day of measurement, and age2 is the age squared) with the corresponding regression coefficients, $b_{1}$ and $b_{2}$ ; F is the regression of the inbreeding coefficient on meat yield with the corresponding regression coefficient, $b_{3}$ ; sex ${}_{j}$ is the j th gender effect ( j = 2, 1: male, 2: female); freq ${}_{k}$ is the k th effect of the number of individuals at birth ( k = 4, 1: singleton, 2: twins, 3: three lambs, 4: four lambs); breeder ${}_{l}$ is the l th effect of the breeder ( l = 78); month ${}_{m}$ is the m th effect of the month of birth ( m = 1–12, January–December); year ${}_{n}$ is the n th effect of year of birth ( n = 25, 1997–2021); and $e_{ijklmn}$ is the random residual error. The model included age at 100 d for Weight100, MLD, fleshiness, and BackFat, ranging from 70–130 d. The animals' ages ranged from 131 to 700 d when evaluating the effect on Weight300 and fleshiness.

The level of significance for accepting the alternative hypothesis about the influence of the inbreeding coefficient on meat yield parameters, as well as for including the effects in the model equation, was p<0.05 . Based on histogram analysis, the evaluated meat yield parameters had an approximately normal frequency distribution. The chosen model's suitability was evaluated using Q–Q plots of residual errors, a plot of standardized residuals and fitted values, and a diagnostic diagram of Cook's distance. The coefficient of determination $R^{2}$ ranged from 0.21 for fleshiness to 0.45 for weight at bonitation.

## 3 Results

The Zwartbles breed has been bred in the Czech Republic since 1995 when animals were imported for breeding for the first time, and subsequent years saw further imports from various countries and breeders. As a result, only a minor amount of inbreeding is observed in the early years of Zwartbles breeding in the Czech Republic.

**Table 1:** Descriptive statistic for the coefficient of inbreeding ( F ) for the basic dataset.

N	Mean	Minimum	Maximum	SEM
13 150	3.64	0.00	42.37	0.0406

SEM: standard error of the mean.

In the entire Zwartbles population, the average inbreeding coefficient (Table 1) is 3.64 %, with a maximum value of 42.37 %. The observed set of sheep contained 13 150 individuals, which were included in the original set for calculating the amount of inbreeding.

**Table 2:** Frequency of animals in each group of inbreeding coefficients ( F ).

F class	Range of F	Percent		
1	0 %–5 %	72.86		
2	5.1 %–10 %	19.32		
3	10.1 %–25 %	6.67		
4	> 25.1 %	1.16		

The Zwartbles population was divided into four groups based on the level of inbreeding coefficient (Table 2): 0 %–5 %, 5.1 %–10 %, 10.1 %–25 %, and more than 25 %. A total of 72.86 % of animals belonged to the lowest group, while 1.16 % belonged to the highest group.

**Table 3:** Frequency of animals in each group of inbreeding coefficients ( F ) according to birth year.

Birth year	F class			
	1	2	3	4
1997	43			
1998	72			
1999	96			
2000	119		2	
2001	163	15	1	
2002	189	22	3	3
2003	382	44	13	2
2004	445	49	11	1
2005	552	24	24	2
2006	547	35	41	7
2007	554	57	33	8
2008	449	71	31	4
2009	432	58	42	5
2010	335	65	57	6
2011	404	144	37	
2012	497	151	32	4
2013	493	162	23	3
2014	438	161	42	5
2015	471	162	57	12
2016	626	188	53	23
2017	586	238	81	15
2018	554	267	83	13
2019	432	290	85	6
2020	389	189	67	21
2021	307	148	59	12

**Figure 1:**
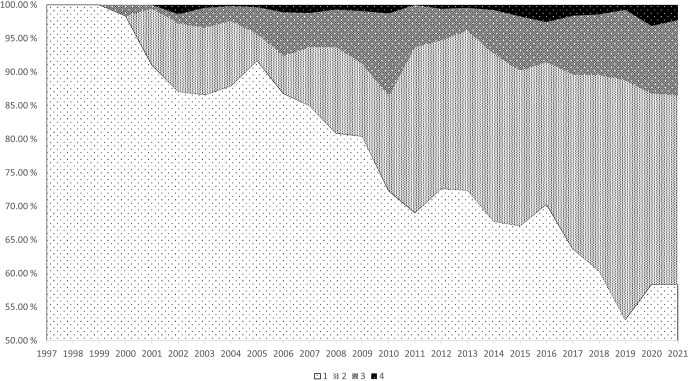
Percentual frequencies of animals in each inbreeding coefficient group according to the birth year. F classes: 1 (0 %–5 %), 2 (5.1 %–10 %), 3 (10.1 %–25 %), and 4 ( > 25 %).

In terms of population development based on individual years of birth (Table 3), there is a steady increase in the rate of kinship. There were only animals in the lowest category of the inbreeding coefficient (0 %–5 %) in the population until the year of birth in 2000, when due to the spread of the breed in Czech farms, animals with a higher proportion of inbreeding began to increase. Since 2015, there have been more than 10 individuals in the set with an inbreeding coefficient greater than 25 %. Figure 1 shows that as the year of birth increases, there is a noticeable increase in inbreeding in this population. According to this, the greatest increase occurred in the second category of the inbreeding coefficient (5.1 %–10 %), which has included roughly 30 % of all animals in the population in recent years. However, more than half of all animals have an inbreeding coefficient of up to 5 %, and only a small percentage have an inbreeding coefficient of more than 25 %.

**Figure 2:**
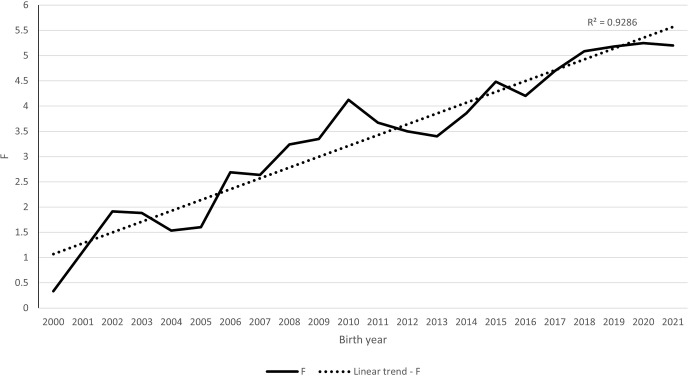
Trend of average coefficient of inbreeding ( F ) in the Zwartbles population.

Figure 2 depicts the development of the inbreeding coefficient in the population by year of birth. A linear function with an average annual increase of 0.2 percentage points can explain 93 % of this trend. In the final years of birth, the inbreeding coefficient increased from nearly zero to more than 5 %.

The influence of the inbreeding coefficient on meat yield parameters was analyzed. The impact on the following meat productivity indicators was assessed: weight at 100 d (Weight100) in kilograms, weight at bonitation (Weight300) in kilograms, *longissimus* *dorsi* muscle thickness (MLD) in millimeters, back fat thickness (BackFat) in millimeters, and fleshiness (Fleshiness) in points according to EUROP. The average age at Weight100 was 101.36 d. The average age of animals at bonitation (Weight300) was 288.19 d. Table 4 shows the average parameters of meat productivity according to the level of the inbreeding coefficient. In the case of animal weight at 100 d, there is a clear decrease in live weight with increasing inbreeding coefficient class. Individuals with an inbreeding coefficient greater than 25 % have a live weight that is more than 2 kg lower than the other groups. This trend, however, is no longer visible in the weight of animals at bonitation (Weight300), where the lowest weight is found in animals with the lowest inbreeding coefficient of up to 5 %. Lower values for MLD thickness can be observed in animals with an inbreeding coefficient greater than 10.1 % compared to animals with an inbreeding coefficient of less than 10 %. The same is true for back fat, where the first two inbreeding classes outperform the classes with a higher inbreeding coefficient. This also holds true for fleshiness, which reaches a value of 0.46 for the lowest inbreeding coefficient ( F ) class and 0.56 for the second F class, while F class 3 reaches a value of 0.26, and class 4 reaches a higher value of 0.36.

**Table 4:** Mean meat parameters according to inbreeding coefficient ( F ) classes.

F class	Weight100 ( n = 11 534)		Weight300 ( n = 2813)		MLD ( n = 11 533)		BackFat ( n = 1731)		Fleshiness ( n = 1715)	
	Mean	SEM	Mean	SEM	Mean	SEM	Mean	SEM	Mean	SEM
1 (0 %–5 %)	30.01 ${}^{a}$	0.0820	52.72 ${}^{b}$	0.1929	23.93 ${}^{a}$	0.1139	2.86 ${}^{a,b}$	0.0240	3.61 ${}^{a}$	0.0273
2 (5.1 %–10 %)	30.00 ${}^{a}$	0.1560	53.91 ${}^{a}$	0.3401	23.91 ${}^{a,b}$	0.5883	2.89 ${}^{a}$	0.0368	3.49 ${}^{a}$	0.0462
3 (10.1 %–25 %)	29.77 ${}^{a}$	0.2669	53.56 ${}^{a,b}$	0.5654	22.26 ${}^{b}$	0.4590	2.66 ${}^{a,b}$	0.0776	2.91 ${}^{b}$	0.1051
4 ( > 25 %)	27.50 ${}^{b}$	0.5728	54.95 ${}^{a,b}$	2.1936	22.81 ${}^{a,b}$	0.9627	2.31 ${}^{b}$	0.1505	3.38 ${}^{a,b}$	0.2562

SEM: standard error of the mean. ${}^{a,b}$ Different superscripts indicate statistically significant differences between means within each factor (rejecting H0 at p<0.05 ).

The inbreeding coefficient (Table 5) had a statistically significant influence on weight at 100 d ( p=0.0007 ), as well as the MLD area ( p=0.0303 ) and back fat ( p=0.0293 ). The effect is highly statistically significant for weight at 100 d but not so much for MLD area and back fat, where the p value ranges from 0.01 to 0.05.

The null hypothesis could not be rejected in the case of weight at bonitation and fleshiness because the inbreeding coefficients have no discernible effect on these parameters. In the case of weight during bonitation, p=0.4587 , and in the case of fleshiness, p = 0.0958.

**Table 5:** Regression of the inbreeding coefficients ( F ) on meat parameters.

Trait	b	p value	$R^{2}$
Weight100	- 0.0602	0.0007	0.2867
Weight300	0.0334	0.4587	0.4496
MLD	- 0.0632	0.0303	0.3707
BackFat	- 0.0132	0.0293	0.2936
Fleshiness	- 0.0127	0.0958	0.2143

b : regression coefficients of F on meat traits. p value: significance of regression coefficient in the GLM. $R^{2}$ : coefficient of determination of the GLM.

The regression coefficient for weight at 100 d reaches - 0.0602, implying that a 1 % increase in the inbreeding coefficient results in a 60.2 g drop in live weight at 100 d. When the inbreeding coefficient increases by 1 %, the MLD thickness decreases by 0.06 mm on average, and the back fat decreases by 0.013 mm. The weight during bonitation is not significantly affected by the degree of inbreeding, and the same applies to fleshiness; the regression coefficients can be considered zero. However, in the case of fleshiness, where the null hypothesis was rejected at the level of p=0.0958 , a negative trend is also visible in relation to the level of the inbreeding coefficient.

## 4 Discussion

The overall value of the inbreeding coefficient in sheep populations varies greatly across countries. For example, the average inbreeding coefficient in the Dorper sheep breed in Brazil is 0.32 % (Kiya et al., 2019). In contrast, for the Morada Nova breed, the authors report an average value of 2.88 % (Matos and Lôbo, 2021). Another study on two breeds in Italy found a large variation in the inbreeding coefficient in different sheep populations, with one breed having an inbreeding value of 5.3 % and the other having an inbreeding value of 15.3 % (Cesarani et al., 2023). An inbreeding analysis of three meat breeds in Denmark showed variability in the inbreeding coefficient ranging from 1.2 % to 2.6 %, with an intergenerational increase in inbreeding of around 1 % (Norberg and Sorensen, 2007). The different values of the inbreeding coefficient in different populations can be attributed to reproduction management, i.e., the different intensity and time of use of the stud in breeding. The average value of the inbreeding coefficient in our study is 3.64%, which is within the range of average values compared to other populations. Although this coefficient is much higher than for extensively bred breeds (Kiya et al., 2019), it also reaches much lower values than for intensively bred breeds (Cesarani et al., 2023). It is true that the above-mentioned values of the inbreeding coefficient result in great variability between populations, which is also due to different mating management and different ways of using studs in herds. From this point of view, the level of inbreeding in the Zwartbles population in the Czech Republic can be assessed as satisfactory. Also, the annual average increase in the inbreeding coefficient is not as significant as stated, for example, by Norberg and Sorensen (2007).

In terms of the structure of the inbreeding coefficient in the Lecesse sheep population in Italy, 11.86 % of the animals had an inbreeding coefficient greater than 10 %, 49.15 % had an inbreeding value of 0 %–10 %, and 38.98 % were non-inbred (Selvaggi et al., 2010). The Dorper breed also has the highest number of animals in the 0 %–5 % inbreeding category and the lowest number of animals in the 25 % inbreeding category. This is consistent with the findings in Table 3 and Fig. 1. From a comparison with other authors, the results of Zwartbles in the Czech Republic can be evaluated positively, which also corresponds to the average inbreeding coefficients. Compared to the Lecesse sheep population (Selvaggi et al., 2010), there are many more animals in the 0 %–5% inbreeding category in our population. This may be related to the fact that the Zwartbles breed has only been bred in the Czech Republic since 1997, and the population was based on unrelated animals.

The value of the inbreeding coefficient is also increasing in the Florina sheep population. Its value increased linearly between 1997 and 2017 but then increased even more as a result of scrapie resistance breeding (Tsiokos and Ligda, 2021). A very similar trend of increasing inbreeding coefficients, as shown in Fig. 2, is reported by the authors in a study of kinship in the Marwari breed, where inbreeding increased from zero to more than 2.5 % between 1975 and 2020 (Vyas et al., 2022). The increase in the coefficient is observed similarly in our work, where it shows a linear trend and confirms the increasing coefficient of inbreeding in the population, which is also a noticeable trend in many other pieces of research on various animal species, such as cattle (da Silva et al., 2019) or goats (Paiva et al., 2020).

The influence of the inbreeding coefficient on meat productivity indicators is described not only in sheep but also in pigs (Vígh et al., 2008) or beef cattle (Lozada-Soto et al., 2021). There was a statistically significant effect of the inbreeding coefficient on the development of MLD and the thickness of back fat in the work dealing with the effect of inbreeding on meat yield in the Dorper sheep breed, which corresponds to our results. Conversely, the authors report an inconclusive effect of inbreeding in the Dorper breed for weight in 90 and 100 d, which contradicts our findings. A study in Moghani sheep shows a negative relationship between inbreeding and weight at 90 d, where a 1 % increase in inbreeding resulted in a 7 g reduction in live weight (Dorostkar et al., 2012). A negative effect of inbreeding is also observed for the weight at 90 d in the Guilan sheep breed, with the regression coefficient reaching a value of - 28.406 g (Eteqadi et al., 2014).

A study of three meat sheep breeds, namely the Texel, Shropshire, and Oxford Down breeds, revealed inbreeding depression in an average daily gain of up to 2 months (Norberg and Sorensen, 2007). According to our findings, the inbreeding effect was evident for the weight at 100 d but not for the weight at bonitation. The fact that the growth of the lamb is also influenced by the maternal component in the case of weight at 100 d can also play a role here, where the inbreeding coefficient can also be applied negatively for the mother. A study of the Suffolk breed in Great Britain looks at the relationship between direct and maternal components in lamb growth (Maniatis and Pollott, 2002).

The effect of inbreeding on slaughter parameters, particularly MLD thickness and back fat, is not well described in the literature. A negative influence of the inbreeding coefficient on carcass weight has been described for meat breeds of cattle, for which an increase in the inbreeding coefficient by 1 % results in a decrease in carcass weight from - 0.87 to - 1.90 kg (Mc Parland et al., 2008). The authors of this study also state that in some meat breeds of cattle, poor musculature and development of individual muscle parts were observed in live animals.

Genetic parameters were estimated for Brahman cattle, and the effect of inbreeding on various useful properties was also considered. Among other things, back fat thickness, rib eye area, and rump fat thickness were evaluated. The authors find no evidence that inbreeding has a negative effect on these parameters (Bessa et al., 2021).

The influence of the inbreeding coefficient on the MLD area was demonstrated in Dorper sheep, where a 1 % increase in inbreeding resulted in a 0.0198 cm ${}^{2}$ increase in MLD area (Kiya et al., 2019), which is in contrast to our results, where there was a statistically significant reduction in MLD height. Our findings indicate that the inbreeding coefficient may have a negative effect on slaughter parameters, specifically the thickness of MLD and back fat; therefore, studies should focus on these qualitative meat yield parameters, as they are important and useful. These parameters were evaluated in Zwartbles sheep in the Czech Republic (Komprda et al., 2012). The significance of these qualitative parameters is described in a study on a nucleus herd of meat sheep in Norway that dealt with meat performance parameter selection (Kvame and Vangen, 2007). The influence of the inbreeding coefficient on meat yield parameters generally corresponds to the results of other authors. Our results show a nonsignificant effect on fleshiness, but the p value is only 0.09, so a certain negative relationship between fleshiness and inbreeding can be observed here as well. There was also a nonsignificant effect on weight at bonitation (Weight300), where the animal's growth has been influenced by external factors for a long time.

## 5 Conclusions

The most important production characteristic of Zwartbles sheep is meat yield. If the current trend of increasing inbreeding continues, there is a risk of decreased productivity in the future. Because inbreeding is constantly increasing, it is necessary to eliminate the reproduction of individuals in group 4 in order to prevent genetic defects and a decrease in productivity. Such animals should be removed from breeding, or their offspring should not be allowed to reproduce. Simultaneously, blood exchange and inbreeding reduction using newly imported studs are appropriate in the near future. Another option for reducing inbreeding would be to also determine parentage in ewe lambs as part of a more accurate pedigree control.

The research has also shown that if the inbreeding values increase, there is a reduction in MLD and back fat. Inbreeding also has a negative impact on weight at 100 d. However, because an individual's body weight at bonitation is influenced by more external factors, the influence of inbreeding on weight at 300 d is inconclusive.

## Data Availability

Data are available from the corresponding author upon request.
